# Rare and Serious Adverse Effects of Anti-Tumor Necrosis Factor-Alpha (TNF-α) Agents in Crohn’s Disease

**DOI:** 10.7759/cureus.14768

**Published:** 2021-04-30

**Authors:** Sun Kim, Peter Edelstein

**Affiliations:** 1 Colorectal Surgery, University of Central Florida College of Medicine, Orlando, USA; 2 Colorectal Surgery, University of Central Florida, Orlando, USA

**Keywords:** crohn’s disease, tnf-alpha, adverse effects, inflammatory bowel disease, adalimumab, hemorrhage, white matter demyelination

## Abstract

Crohn’s disease patients experience a higher rate of postoperative complications than do general surgical patients. The use of anti-tumor necrosis factor-alpha (anti-TNF-α) therapy in the treatment of severe Crohn’s disease and other autoimmune inflammatory conditions is increasing and expanding. We describe the case of a 47-year-old female Crohn’s patient who experienced two rare and serious adverse effects of anti-TNF-α therapy following laparoscopic ileocectomy for obstructive Crohn’s disease. On the first postoperative day, the patient developed intra-abdominal hemorrhage requiring transfusion and emergency abdominal exploration. Findings were consistent with a rare hemorrhagic complication of her anti-TNF-α medication. She recovered and was ultimately discharged from the hospital without further complications. Less than 24 hours following discharge, the patient suffered two grand mal seizures. Imaging demonstrated white matter demyelination of the brain. The patient recovered, again, with no clinical sequelae. These two dangerous events are known to rarely be associated with the use of such biologic agents. We review both the therapeutic actions of these medications and the theorized etiologies for these two rare adverse events. Ultimately, this patient’s complications should serve as a cautionary tale in the use of such therapeutics, as well as a reminder of the risks associated with anti-TNF-α use.

## Introduction

Crohn’s disease is an idiopathic inflammatory bowel condition that can affect the entire gastrointestinal tract from the mouth to the anus and is characterized by transmural inflammation and “skip lesions.” A chronic disease, Crohn’s has an annual incidence ranging between three and 20 cases per 100,000 people [1]. Most Crohn’s disease patients will require at least one surgical bowel resection during their lifetime. Although surgery improves their quality of life, the majority of patients will suffer disease recurrence, and a reoperation rate of 50-60% is generally reported [2].

Crohn’s disease patients experience a higher rate of postoperative complications than do general surgical patients. Thus, there has been significant research and advancement in the development of effective prophylactic medical therapy to decrease the incidence of bowel resection in Crohn’s disease patients. Peri-operative steroids are commonly used to address and prevent bowel inflammation, but their success is limited, particularly in patients with clinically aggressive disease. Immunosuppression using azathioprine has demonstrated efficacy in inducing disease remission and can even resolve severe postoperative disease recurrence [2]. There is also evidence that in specific settings, metronidazole can prevent postoperative fistula formation [3]. Finally, TNF-α monoclonal antibodies, such as infliximab and adalimumab, represent next-generation immunomodulators and have demonstrated significant benefits in Crohn’s patients with refractory luminal and fistulizing Crohn’s disease. Early studies have demonstrated the benefit of immediate post-operative administration of biologic therapy in preventing postoperative Crohn’s disease recurrence and fistula formation [4]. A recent meta-analysis showed that anti-TNF monotherapy has been associated with the largest decrease in disease recurrence in patients at moderate or high risk for early recurrence after surgical resection for Crohn’s disease, especially compared to antibiotics, probiotics, budesonide, and aminosalicylates [5].^ ^The Prevention of Recurrence in Crohn’s Disease Patients Undergoing Surgical Resection Who Are at an Increased Risk of Recurrence (PREVENT) study showed significantly decreased rates of recurrence as shown endoscopically at 76 weeks among patients treated with Remicade® (infliximab) compared to placebo (22.4% vs 51.3%; P < .001) [6]. However, these powerful biologic agents carry significant adverse event risks for patients [7], including demyelinating disease, lymphomas, lupus-like syndromes, and congestive heart failure [4].

## Case presentation

A 47-year-old female with a past medical history significant for severe bilateral inguinal hidradenitis suppurativa and Crohn’s disease presented for the evaluation of a clinically symptomatic terminal ileal stricture. Preoperatively, she was taking Humira® (adalimumab; AbbVie, Inc.) injections every other week, and while this did not appear to impact her stricture symptoms, her inguinal hidradenitis suppurativa remained in remission with this treatment. After evaluation and discussion, the patient underwent a laparoscopy, during which she was found to have grossly normal large and small bowel, with the exception of a 20 cm segment of grossly narrowed, hyper-vascularized terminal ileum, which was partially obscured by creeping fat, and minimally dilated ileum proximal to the obstruction. A laparoscopic ileocectomy with primary anastomosis was performed. The patient tolerated the procedure without complication, was extubated in the operating room, and was transferred to the surgical floor following recovery. Pathology demonstrated both active and chronic Crohn’s disease with significant obstruction of the ileal lumen.

On the first postoperative day, the patient’s hematocrit began to drop, and that evening, her systolic blood pressure dropped to 100 mmHg. On examination, her abdomen was moderately distended. The patient was transfused a single unit of packed red blood cells and returned emergently to the operating room for a presumed diagnosis of active hemorrhage. Exploration was performed through a limited midline incision. Upon entering the abdominal cavity, a large volume of clotted and hemolyzed blood was encountered and removed. The abdomen was irrigated and explored in a systematic fashion to identify any source of the hemorrhage. We found no surgical source of the bleeding, noting diffuse, low-rate bleeding from areas of prior surgical dissection, including the right lower quadrant peritoneum and divided peritoneal edges. We also noted diffuse, low-rate oozing from areas where no surgical manipulation had been performed, including both fallopian tubes and the peritoneum overlying the hepatic flexure mesentery.

As there was no role for electrocautery or suture ligation, we irrigated with warm normal saline, packed with laparotomy pads, and transfused two units of fresh frozen plasma (FFP) while warming the patient. The packing was removed, and little bleeding was now identified. Several sheets of SURGICEL and SURGICEL powder (Ethicon US, LLC, Cincinnati​, OH) were placed over areas of the most active diffuse bleeding. Intraoperative blood tests failed to identify any coagulation abnormalities or evidence of disseminated intravascular coagulopathy.

The patient was extubated and transferred to the intensive care unit. Her subsequent recovery was rapid and unremarkable, with no further evidence of bleeding. She was discharged from the hospital nine days after her initial surgery.

**Figure 1:**
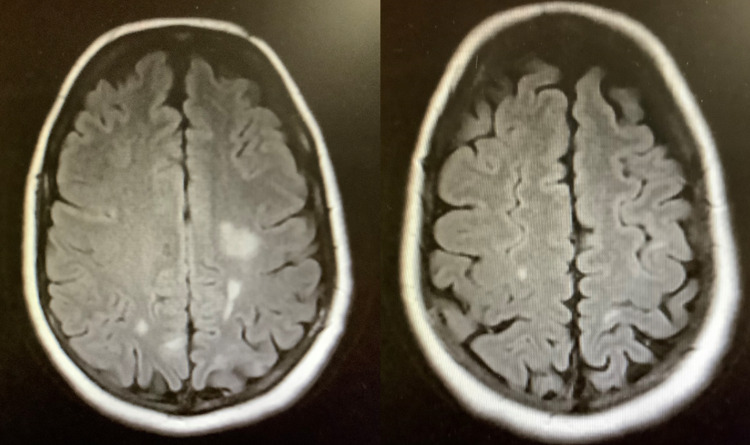
Brain MRI demonstrating subcortical hyperintense lesions of the bilateral parietal lobes consistent with demyelination

Less than 24 hours after hospital discharge, the patient’s husband brought her to the emergency department, stating that following dinner, she suddenly began “acting and talking strangely,” after which he witnessed her suffering a seizure. Soon after presenting, the patient had a second grand mal seizure in the emergency department. A non-contrast head CT was unremarkable. The patient was admitted, treated with standard anti-seizure medications, and suffered no more seizures, recovering rapidly (including a return to normal, oriented, appropriate mental status). MRI of the brain the next day (Figure 1) identified white matter demyelination sites, which were confirmed by neurology to be the likely source of her new-onset seizures.

## Discussion

Both intra-cavitary hemorrhage (including intra-abdominal) and seizures resulting from white matter demyelination are rare but documented adverse effects of adalimumab (Humira®), a TGF-α monoclonal antibody used to treat autoimmune conditions including Crohn’s disease [8].

TNF-α is a pro-inflammatory cytokine that modulates inflammation by inducing the cytokines IL-1 and IL-6, activating neutrophils and enhancing leukocyte migration [9]. TNF-α is expressed as a soluble cytokine (sTNF) via the cleavage of tmTNF by TNF-α converting enzyme (TACE). sTNF binds to TNFR1 and mediates apoptosis and chronic inflammation, and tmTNF binds TNFR2 and activates genes important for cell survival, inflammation resolution, myelination, and remyelination by promoting the proliferation of oligodendrocyte precursor cells [10].

TNF-α blockers are theorized to cause demyelination of the white matter of the brain by downregulating TNFR2 receptors, which are needed for oligodendrocyte precursor proliferation and repair of myelin [10]. These agents may also alter cellular responses to cytokines by upregulating IL-12 and IFN-γ and downregulating IL-10, resulting in a disease condition similar to multiple sclerosis [11]. Finally, TNF-α blockers have the potential to unmask an underlying latent infection, which may lead to autoimmune demyelination. Interestingly, susceptibility to multiple sclerosis is associated with a single nucleotide polymorphism at the TNFSF1A gene that causes the expression of a soluble form of TNFR1 that inhibits TNF-α in humans. Thus, an intrinsic or extrinsic TNF-α blockade may cause white matter demyelination. Extrinsic anti-TNF-α therapy may be discontinued with a complete reversal of symptoms, thus differing pathophysiological from multiple sclerosis, which is progressive and with permanent effects. Four cases of central nervous system (CNS) demyelination in patients treated with anti-TNF-α therapy have been documented, with evidence of full resolution of symptoms upon discontinuation of the offending drug [12]. At this early post-event stage, our patient represents the fifth documented case of demyelination with clinical resolution.

Hemorrhagic complications associated with adalimumab therapy have been documented, with one study suggesting that patients may develop an acquired factor XI deficiency leading to increased risk of bleeding [13]. Surgery can trigger excessive bleeding in severe Factor XI deficiency. Furthermore, there have been several documented case studies of patients treated with adalimumab who have developed acquired hemophilia A, a rare and life-threatening coagulopathy characterized by spontaneous hemorrhage in patients with no history of prior bleeding. In another case report, a patient experienced extensive subcutaneous bleeding three years after the initiation of adalimumab treatment for necrotizing scleritis [14].^ ^Due to cytokine inhibition by a TNF-α blockade, it is suggested that autoreactive T cells are instead able to be activated, triggering a humoral antibody response to self-antigens, such as coagulation factors, rather than being in a state of immunogenic tolerance [12].

Our patient suffered from both obstructive terminal ileal Crohn’s disease and severe hidradenitis suppurativa, the latter condition well-controlled with bi-weekly Humira® administration. In the immediate postoperative period, she suffered from two adverse events associated with the use of this biologic agent. Her diffuse hemorrhage, a rare complication of adalimumab therapy, appeared to be triggered by her surgical procedure. Fortunately, this resolved with transfusion of coagulation factors and use of intra-abdominal compression and hemostatic agents. Her second drug-related complication, seizures resulting from adalimumab-associated brain white matter demyelination, is extremely rare. Fortunately, she has experienced no subsequent seizures since her initial presentation, and she remains on anti-seizure medication. The patient is no longer on Humira®, necessitating alternative therapy for both her Crohn’s disease and hidradenitis suppurativa.

## Conclusions

This appears to be the first case report documenting two rare and serious adverse effects of TNF-α antibody therapy in a single patient following surgery for Crohn’s disease. Adalimumab appears to have the potential to stimulate the production of autoantibodies against coagulation factors, risking hemorrhage. In addition, a TNF-α blockade may cause white matter demyelination by inhibiting oligodendrocyte precursor proliferation. It is unclear why this patient is particularly susceptible to differing adverse event pathways leading her to suffer two seemingly unrelated, rare, and serious adverse effects. As the use of anti-TNF-α therapy in the treatment of autoimmune inflammatory conditions increases and expands, and with the clear acknowledgment that the use of such agents is immensely beneficial in managing many patients’ severe, refractory Crohn’s disease, our patient’s experience should serve as a warning as to the rare yet serious risk of biologic agent use and as a guide should unanticipated and even seemingly unrelated complications occur in patients undergoing anti-TNF-α treatment.
